# Supplementary material for: Risk factors for microbiologic failure in children with Enterobacter species bacteremia
Source: PLoS One. 2021 Oct 7;16(10):e0258114. doi: 10.1371/journal.pone.0258114 (PMC8496803; doi:10.1371/journal.pone.0258114)
Supplement: S1 Table — CLABSI (Central-line associated bloodstrea infection); CVC (central venous catheter); mo (months); VAP (ventilator associated pneumonia); SSI (surgical site infection); RV (right ventricle); PA (pulmonary artery). (DOCX) [file pone.0258114.s001.docx]

**Supplemental Table 1. Demographic and clinical characteristics of pediatric patients with microbiologic failure following *Enterobacter spp.* bacteremia**

| Case no. | Age | Sex | Underlying condition | Primary site of infection | Empiric antibiotic therapy | Definitive antibiotic therapy | Initial isolate susceptible to ceftriaxone | Time to subsequent positive culture from initial bacteremia episode (days) | Source of subsequent culture | *Species* | Relapse or persistence of infection | Development of resistance to ceftriaxone | Time to source control (days) | Outcome | Comments |
| --- | --- | --- | --- | --- | --- | --- | --- | --- | --- | --- | --- | --- | --- | --- | --- |
| 1 | 4 years | F | malignancy (brain tumor) | CLABSI | ceftriaxone | piperacillin-tazobactam; ceftriaxone | Yes | D+3 | blood | *E. cloacae* | Relapse | No | 6 | Survived | CVC (port removed) due to relapse |
|  |  |  |  |  |  |  |  | D+6 | blood |  |  |  |  |  |  |
| 2 | 1 years | F | prematurity;  pulmonary  hypertension | blood | ceftriaxone | cefepime | Yes | D+21 | tracheal aspirate | *E. cloacae* | Relapse | Yes | N/A | Survived | Treated as VAP due to increase in ventilator support and focal infiltrate on imaging |
| 3 | 2 weeks | F | prematurity;  necrotizing enterocolitis | intra-abdominal infection | ceftriaxone and gentamicin | cefotaxime | Yes | D+3 | blood | *E. cloacae* | Persistence | Yes | 3 | Died on D+7 | Intraabdominal wash-out and bowel resection, continued to have multiple intraabdominal abscesses |
|  |  |  |  |  |  |  |  | D+5 | blood |  |  |  |  |  |  |
| 4 | 2 years | F | liver transplant | biliary | ciprofloxacin | cefotaxime; meropenem | Yes | D+4 | peritoneal fluid | *E. cloacae* | Relapse | No | 4 | Survived | Complicated intraabdominal and biliary infection; drained but reaccumulated |
|  |  |  |  |  |  |  |  | D+22 | peritoneal fluid and blood |  |  |  |  |  |  |
|  |  |  |  |  |  |  |  | D+30 | bile and blood |  |  |  |  |  |  |
| 5 | 2 years | F | malignancy (AML) | CLABSI | piperacillin-tazobactam | ceftazidime; piperacillin-tazobactam | Yes | D+4 | blood | *E. cloacae* | Persistence | No | 4 | Survived | CVC removed due to persistence |
| 6 | 16 years | F | cloacal anomaly | SSI | gentamicin | cefepime and gentamicin; ceftriaxone | Yes | D+5 | wound (hip abscess) | *E. cloacae* | Relapse | No | 4 | Survived | Hip wound incision and drainage after fevers; treated for pelvic osteomyelitis |
| 7 | 7 years | M | short gut syndrome | CLABSI | piperacillin-tazobactam | ceftriaxone | Yes | D+3 | blood | *E. cloacae* | Persistence | No | 5 | Survived | CVC removed due to persistence |
|  |  |  |  |  |  |  |  | D+4 | blood |  |  |  |  |  |  |
| 8 | 6 mo | M | liver failure; biliary atresia | CLABSI | piperacillin-tazobactam | ceftriaxone | Yes | D+22 | blood | *E. asburiae* | Relapse | Yes | 11 | Survived | CVC exchanged on D+11 |
| 9 | 4 mo | M | prematurity; gastroschisis | CLABSI | ceftriaxone and gentamicin | Cefotaxime + gentamicin | Yes | D+3 | blood | *E. aerogenes* | Relapse | No | 3 | Survived | CVC removed |
| 10 | 9 mo | M | prematurity; short gut syndrome | CLABSI | piperacillin-tazobactam | Piperacillin-tazobactam | Yes | D+20 | urine | *E. cloacae* | Relapse | No | 3 | Survived | CVC removed |
| 11 | 10 mo | F | liver transplant | intra-abdominal infection | meropenem | meropenem | No  (MIC >64) | D+21 | peritoneal fluid | *E. cloacae* | Relapse | N/A (initial isolate resistant) | 2 | Survived | Peritoneal washout and drain placement |
| 12 | 3 years | F | DiGeorge syndrome; congenital heart disease; ventilator dependent | endocarditis of prosthetic material | ceftriaxone and gentamicin; cefepime | Meropenem + FQ; meropenem + gentamicin | No  (MIC >16) | D+3 | blood | *E. cloacae* | Relapse | N/A (initial isolate resistant) | 4 | Survived | RV to PA conduit replacement due to persistence, septic emboli |
|  |  |  |  |  |  |  |  | D+25 | body fluid (splenic abscess) |  |  |  |  |  |  |

CLABSI (central-line associated bloodstream infection); CVC (central venous catheter); mo (months); VAP (ventilator associated pneumonia); SSI (surgical site infection); RV (right ventricle); PA (pulmonary artery)
