# Supplementary material for: Risk factors for microbiologic failure in children with Enterobacter species bacteremia
Source: PLoS One. 2021 Oct 7;16(10):e0258114. doi: 10.1371/journal.pone.0258114 (PMC8496803; doi:10.1371/journal.pone.0258114)
Supplement: Attachment — Submitted filename: Response to Reviewers.docx [file pone.0258114.s002.docx]

Iddya Karunasagar, PhD

Academic Editor

PLOS ONE

August 19, 2021

Dear Dr. Karunasagar,

We thank you for inviting us to submit a revised version of our manuscript entitled, “Risk factors for microbiologic failure in children with *Enterobacter* species bacteremia” for publication in *PLOS* ONE. We are grateful to you and the reviewer for taking the time to provide us with useful and constructive feedback on our manuscript. We have taken all of the recommendations provided into consideration and have made substantial changes to our manuscript, which we believe significantly strengthen our paper. We hope that these revisions and the responses provided address all the issues and concerns you and the reviewer have raised. Below is a point-by-point response to the questions and comments enumerated in your decision letter.

**Editor comments:**

1. **Please ensure that your manuscript meets PLOS ONE’s style requirements, including those for file naming.**

Thank you for your comment and including the relevant links for PLOS ONE’s formatting templates. These templates and the formatting requirements have been reviewed in detail to ensure our manuscript meets the style requirements. The supplemental table file name has been updated in accordance with these requirements. File names for the components of the resubmission have been named according to the recommendations outlined on the PLOS ONE website for Revising Your Manuscript.

2. **Please include captions for your Supporting Information files at the end of your manuscript, and update any in-text citations to match accordingly.**

Thank you for pointing out this omission. A caption for Supplemental Table 1 has also been added at the end of the manuscript body under the level 1 heading “Supporting information,” lines 477-480. In-text citations for this supplemental information were verified to comply with the Journal’s formatting requirements.

3. **Thank you for including your ethics statement on the online submission form: "This study was approved by Baylor College of Medicine’s Institutional Review Board. Approval number H-42231. A waiver of consent was granted by the Institutional Review Board given the retrospective nature of the study and minimal risk to participants.". To help ensure that the wording of your manuscript is suitable for publication, would you please also add this statement at the beginning of the Methods section of your manuscript file.**

Thank you for bringing this to our attention. The wording above has been added to the first paragraph of the Methods section, lines 97-100.

**Reviewer 1 comments:**

1. **How was ESBL and AmpC detection done in the study? The authors explain 3rd gen cephalosporins but do not explain which 3rdgen cephalosporin was used for testing or treatment. What was the general percentage of AmpC in the hospital? Did that change over the study period?**

We appreciate Reviewer 1’s comments. We agree verifying the mechanism of resistance would be interesting. However, genotyping of Amp-Cs is not routinely performed for clinical

specimens at our institution in accordance with CLSI guidelines. Additionally, as *Enterobacter* species chromosomally encode an inducible AmpC beta-lactamase, the potential for AmpC-mediated resistance was assumed for all *Enterobacter* species. A sentence clarifying this has been added to the methods section in lines 110-112. Similarly, we are not able to estimate the percentage of AmpC producing organisms or ESBLs in our hospital or how this may have changed over time as testing is not routinely performed by the clinical microbiology laboratory. An acknowledgment and explanation of this limitation has also been added to the discussion section, lines 356-365.

We agree the term “third-generation cephalosporins” could cause confusion as different institutions use various agents of this antibiotic class and some agents are no longer available (i.e, cefotaxime). Antibiotic susceptibility testing for all 3^rd^ generation cephalosporins was inferred from the results of ceftriaxone testing and a sentence clarifying this has been added to the methods section, line 108. A footnote “c” has also been added to Table 1 to indicate that third-generation cephalosporins refers specifically to ceftriaxone, cefotaxime or ceftazidime, line 259. Column headings in Supplemental Table 1 have also been updated to specify whether the initial isolate was susceptible to ceftriaxone rather than “3GCs” and, similarly, whether there was development of resistance to ceftriaxone rather than “3GCs.”

2. **The methodology defines including all cases less than 21 years who developed infection with Enterobacter species. The supplementary table shows most cases (except 3 cases) in children less than 4 years. What was the possible source for the organism. Was there a possibility of an outbreak with more than 2 cases occurring around the same time? Or was there a constant reservoir in the hospital?**

We agree with Reviewer 1 that these are interesting questions. Prior pediatric studies have noted that young age, chronic medical comorbidities and critical illness are risk factors for *Enterobacter* bloodstream infections. A sentence regarding this observation has been added to the introduction, lines 64-66. Similarly, the majority of our cohort included younger patients <5 years with chronic medical comorbidities and a comment highlighting this has been added to the discussion, lines 292-294. The source of the organism is unclear though the majority of patients had healthcare-associated infections. Only 2 of the cases with microbiologic failure occurred around the same time, though this was in different hospital units making an outbreak unlikely. Cases were otherwise evenly distributed over the 7-year study period and a sentence clarifying this has been added to the results section, line 214. Where isolates were available for genotyping among case patients, there was considerable interpatient strain genetic variability suggesting a lack of a common source.

3. **Since most were associated with CLABSI, what were the infection control measures put in place to reduce the occurrence?**

We thank Reviewer 1 for their comment. This is an interesting question but, unfortunately, we did not have the data to be able to comment on whether any infection prevention measures may have differed between cases and controls. However, CLABSI prevention has been a focus in our hospital, as it has been nationally, and numerous measures (e.g., insertion bundles, handwashing campaigns, etc.) have been instituted over time, though this would not be expected to have differed between cases and controls. A comment to this effect was added to the discussion, lines 367-370.

4. **What was the antibiogram of the hospital and what was the basis of the choice of empirical antibiotics described in the study? What was the data on escalation and de-escalation of antibiotics and did they impact outcomes?**

Seventy-seven percent of patients had an isolate that was considered “susceptible” to 3^rd^ generation cephalosporins at baseline based on CLSI breakpoints. The heterogeneity of empiric antibiotic regimens, like definitive therapy, makes it difficult to determine if any individual agent was associated with worse or better outcomes as acknowledged in the discussion. We note that the vast majority of patients in both groups (92% cases vs. 89% controls) received “appropriate” empiric therapy as defined by an agent given by the correct route (i.e., parenterally), at the correct dose, and for which the isolate was ultimately identified as susceptible in retrospect based on antimicrobial susceptibility results. A column describing empiric therapy for cases has been added to Supplemental Table 1. Similarly, definitive therapy was deemed appropriate in 100% of cases and 97% of control patients. We also note that definitive antibiotic regimens were similar between the case and control groups, though in both groups narrower antimicrobial agents could have been selected in 42% and 50% of cases and controls respectively (i.e., de-escalated) based on susceptibility results alone. It is uncertain if results would have been similar had these patients received narrower spectrum therapy, though our results that relapse was rare in patients treated with third-generation cephalosporins is encouraging that these agents can be safely used instead of cefepime and carbapenems when the isolate is susceptible and source control is achievable.

5. **Table 1 on the demographics and risk factors classifies hepatobiliary disease and intraabdominal/hepatobiliary separately. Is there a reason for the same?**

We thank the Reviewer for pointing out this confusing nomenclature. In Table 1 “hepatobiliary” is listed as an underlying comorbid condition and “intraabdominal/hepatobiliary” is listed as a primary site of infection. The term “hepatobiliary” has been removed from the primary site of infection to avoid confusion and a footnote has been added to indicate that “intraabdominal” as a primary site of infection refers to any deep-seated intraabdominal infection, including hepatobiliary infections such as cholangitis, lines 224-225.

6. **Materials and methods describes genotyping as if done on all isolates. But only 7 isolates were chosen. What was the basis of the choice of isolates?**

Thank you for pointing out this potential inconsistency. The relevant materials and methods section for genotyping has been clarified to indicate genotyping was done only on cases with microbiologic failure of their Enterobacter infection who had both isolates available for genotyping (i.e., the index isolate and the isolate from the relapse of infection), lines 163-168. Genotyping was not done on control patients. Unfortunately, banked specimens were not available from the clinical microbiology laboratory for every case patient to be able to verify relapse of infection with the same strain versus a *de novo* infection. The genotyping section of the methods has also been moved to follow the study design and definitions sections that establish the definitions for cases and controls and microbiologic failure to avoid confusion.

We thank you and the reviewer again for your time and thoughtful comments and the opportunity to strengthen our manuscript based on this feedback. We appreciate your reconsideration of our submission.

Sincerely,

Juri Boguniewicz, MD

Corresponding Author

Assistant Professor of Pediatrics

University of Colorado School of Medicine

Juri.boguniewicz@childrenscolorado.org
